# Health System Factors in Head and Neck Cancer Diagnosis: A HEADSpAcE Consortium Qualitative Study in Glasgow and Montevideo

**DOI:** 10.1002/hed.70262

**Published:** 2026-05-10

**Authors:** Grant Creaney, Iván Lyra González, Mauricio Cuello, Mariél de Aquino Goulart, Alex D. McMahon, Claire Paterson, James McCaul, Maja Popovic, Lorenzo Richiardi, Silvia Lopez de Blanc, Maria Paula Curado, Marta Vilensky, HEADSpAcE Consortium, Elmira Ebrahimi, Apiwat Sangphukieo, Hanla A Park, Valerie Gaborieau, Wolfgang Ahrens, Laia Alemany, Lidia MRB Arantes, Jaroslav Betka, Cristina Canova, David I Conway, Mauricio Cuello, Maria Paula Curado, Ana Carolina de Carvalho, Jose Carlos de Oliviera, Mark Gormley, Maryam Hadji, Claire M Healy, Ivana Holcatova, Luis P Kowalski, Pagona Lagiou, Areti Lagiou, Gary J Macfarlane, Sandra Perdomo, Luis Felipe Ribiero Pinto, Jose Roberto V Podesta, Jerry Polesel, Miranda Pring, Hamideh Rashidian, Ricardo R Gama, Lorenzo Richiardi, Max Robinson, Paula A Rodriguez‐Urrego, Sheila C Soares‐Lima, Nicolas Timpson, Marta Vilensky, Sandra V von Zeidler, Tim Waterboer, Kazem Zendehdel, Ariana Znaor, Paul Brennan, James McKay, Shama Virani, Tom Dudding, Roque Adam, Antonio Agudo, Shaymaa F AlWaheidi, Miquel Angel Pavon, Namrah Anwar, Paola Engelmann Arantes, Lisa Arguello, Yubelly Avello, Lucas Avondet, Ana M Baldión‐Elorza, Camila Batista Daniel, Bianca Beraldi, Barbara Berenstein, Patricia Bernal, Natália Bernardino Rodrigues, Josipa Bilic Zimmermann, Marianna G Botta, Jesús Brenes, Nicole Brenner, Carol Brentisci, Catalina Burtica, María L Cabañas, Erick Cantor, Raiany S Carvalho, Andre L Carvalho, Luigi Chiusa, Priscilia Chopard, Qurratulain Chundriger, Omar Clavero, Isabela Costa, Grant Creaney, Cecilia Cuffini, Tauana C Dias, Evandro Duccini de Souza, Lais C Durant, Alberto Escallón, Gisele Aparecida Fernandes, Béatrice Fervers, Valentina Fiano, Frederico Firme Figueira, Regina Furbino Villefort, Manuela Gangemi, Paolo Garzino‐Demo, Mahin Gholipour, Raul Giglio, Mariel A Goulart, Jéssica Graça Sant'Anna, Marek Grega, Anna Clara Gregório Có, Arnau Guasch, Jose A Hakim, David N Hayes, Marco Homero de Sá Santos, Katrina Hurley, Magalí Insfran, Giuseppe C Iorio, Moghira Iqbaluddin Siddiqui, Jannik Johannsen, Martin Kaňa, Jens Peter Klussmann, Evelio Legal, Jeferson Lenzi, Fernando Luiz Dias, Iván Lyra González, Willene Machado Zorzaneli, Ricardo Mai Rocha, Manel Mañós, Priscila Marinho de Abreu, Maryam Marzban, James McCaul, Alex D. McMahon, Carlos Mena, Elismauro F Mendonça, Laura Mendoza, Lorena Meza, Birgitta Michels, Matinair S Mineiro, Chiara Moccia, Pamela Mongelos, Ana L Montealegre‐Páez, Francisca Morey Cortes, Alvaro Muñoz, Andy Ness, Aline B Neves, Marco Oliva, José Carlos de Oliveira, Hernán Ortiz, José Ortiz, Marta Osorio, Vanessa Ospina, Oliviero Ostellino, Mauricio Palau, Claire Paterson, Sonia Paytubi Casabona, Giancarlo Pecorari, David M Pereira, Olivia Pérol, Shahid Pervez, Alicia Pomata, Maja Popovic, Alisson Poveda, Carol P Prado, Kristina M Prager, Guglielmo Ramieri, Juliana NI Rego, Rui M Reis, Helene Renard, Umberto Ricardi, Giuseppe Riva, Frederic Rodilla, Ingrid Rodriguez, María I Rodríguez, Alastair Ross, Pierre‐Eric Roux, Tazeen Saeed Ali, Pierre Saintigny, Juan J Santivañez, Cristóvam Scapultampo‐Neto, Javier Segovia, Agenor Sena, Ricardo Serrano, Shachi J Sharma, Oliver Siefer, Stephanie Smart, Bruna P Sorroche, Cinthia Sosa, Juliana Souza de Oliveira, Antonella Stura, Steven Thomas, Oscar Torres, Sara Tous, Gonzálo Ucross, Adriana Valenzuela, José Roberto Vasconcelos de Podestá, Alex Whitmarsh, Sylvia Wright, Shaymaa Alwaheidi, Paul Brennan, Shama Virani, Al Ross, David I Conway

**Affiliations:** ^1^ School of Medicine, Dentistry and Nursing University of Glasgow Glasgow UK; ^2^ Glasgow Head and Neck Cancer (GLAHNC) Research Group Glasgow UK; ^3^ Service of Oncology Manuel Quintela Hospital Montevideo Uruguay; ^4^ Beatson West of UK Cancer Centre NHS Greater Glasgow and Clyde Glasgow UK; ^5^ Queen Elizabeth University Hospital NHS Greater Glasgow and Clyde Glasgow UK; ^6^ Cancer Epidemiology Unit, Department of Medical Sciences University of Turin, and CPO‐Piemonte Turin Italy; ^7^ Facultad de Odontología Universidad Nacional de Córdoba Cordoba Argentina; ^8^ A. C. Camargo Centre Sao Paulo Brazil; ^9^ Universidad De Buenos Aires Buenos Aires Argentina; ^10^ International Agency for Research on Cancer (IARC) Genomic Epidemiology Branch Lyon France; ^11^ School of Health, Education, Policing and Sciences University of Staffordshire Stafford UK

**Keywords:** head and neck cancer, health systems, qualitative methods

## Abstract

**Background:**

Head and neck cancer (HNC) is a devastating diagnosis, with advanced‐stage disease leading to poorer outcomes. This qualitative study aimed to identify health system factors associated with stage of HNC diagnosis.

**Methods:**

Qualitative semistructured interviews with HNC patients and clinicians were undertaken in two purposively selected regional cancer centers in Scotland and Uruguay. Transcripts were analyzed thematically via Template Analysis, utilizing conventional cancer diagnostic intervals and a systems engineering model of how patient and organizational outcomes emerge from complex interactions.

**Results:**

Sixteen health system themes and 45 subthemes were identified from 29 interviews. Themes important for timely diagnosis included: public awareness of risk/symptoms; patient ability to access and navigate through HNC pathways; socioeconomic/geographic inequalities; and ways of working between healthcare teams.

**Conclusions:**

Health system factors associated with diagnostic stage were identified across centers and participant groups that could inform service changes to support earlier stage diagnosis of HNC.

**Abbreviations**
GP: general practitioner
HEADSpAcE: Translational Studies of Head and Neck Cancer in South America and Europe
HNC: head and neck cancer
HPV: human papilloma virus
NHS: National Health Service
PCP: primary care practitioner
SEIPS: system engineering initiative for patient safety
TNM: tumor, nodes, metastasis
WOSCAN: West of Scotland Cancer Network

## 1 Background

Head and neck cancer (HNC) is a significant and increasing global public health problem ranking as the seventh highest cancer in incidence, with 600 000 newly diagnosed cases of oral cavity, oropharynx, hypopharynx, and larynx cancer annually, and an estimated 400 000 deaths annually, making HNC the ninth highest cancer in mortality globally [1]. It has been shown that more than half of HNC cases are diagnosed at advanced stage disease [2, 3], defined as stages III and IV according to the TNM Eighth Edition [4]. Diagnosing HNC at an early stage is important for improving outcomes, including treatment‐related morbidity, quality of life and survival [5, 6].

Local and national health systems and infrastructures are involved in diagnosis and treatment of HNC, with both human (specialist multidisciplinary teams) and technical (diagnosis services and equipment) resources being important elements [7]. For HNC diagnosis, tissue biopsy for histopathological specialist analysis remains the gold‐standard, supplemented by imaging and enhanced visualization for clinical staging [8]. Additionally, there is a potential role for a well‐developed, clear care pathway from referral to diagnosis to improve efficiency of diagnosis [9].

Factors that influence the stage at diagnosis of HNC include individual sociodemographic, health and behaviors, and tumor‐related factors such as subsite and HPV positivity associated with oropharyngeal tumors [10]. The role of health systems factors has not been fully investigated for HNC; however, evidence from other solid tumor cancers such as breast, lung, and colon [11, 12, 13], suggests factors such as availability and accessibility of diagnostic facilities are important for stage at diagnosis.

There seems to be an evidence gap in relation to improving the diagnostic care pathway for HNC through detailed study of the role of health system factors. A recently published systematic review on inequalities in advanced stage HNC identified various health system variables of potential interest, but in synthesizing findings focused on individuals' health insurance and ability to access healthcare services [14].

The emerging role of systems science in healthcare services research offers a lens through which a holistic view can be taken and the complexities of delivering good population outcomes addressed [15]. The Head and Neck Cancer in South America and Europe (HEADSpAcE) multicenter consortium [16] provided the context for this study and the opportunity to investigate health systems factors in the stage at diagnosis of HNC by recruiting from within the consortium.

The aim was to identify reported system variables and interactions from patient and staff perspectives to identify areas for future research and service improvement.

## 2 Methods

### 2.1 Setting and Study Design

The setting for this study was two purposively selected regional major cancer centers: The West of Scotland Cancer Network (WOSCAN) in Glasgow, Scotland, and the Hospital de Clinicas in Montevideo, Uruguay. Both are major cancer centers with substantial population coverage. The study was a cross‐sectional qualitative study drawing from theory‐informed, in‐depth, semi‐structured interviews with multiple stakeholders based on nonprobabilistic quota sampling [17]. Sampling, based on the nonhomogenous participant type, underpinning theory, the narrowly defined objectives, and the objective of achieving adequate data to meet the research aims, was set at an overall target of 24 interviews with flexibility to adapt and expand to developing themes from interview data [18, 19]. The centers were selected from the HEADSpAcE Consortium, where they both are the major HNC center in their respective countries; the countries are of similar size and Uruguay was the only high‐income country from South America in the Consortium [20].

### 2.2 Procedures

In order to be eligible, HNC patients had to (a) have been diagnosed with a new, primary HNC in the preceding 2 years; (b) be physically and cognitively able to give informed consent and participate in the study; (c) be at least 18 years of age; and (d) have gone through the diagnostic processes at the respective HNC center.

HNC patient participants were approached in the first instance by local cancer treatment teams and given a high‐level project overview. Those willing to be contacted by the research team were then approached in‐person at a subsequent clinical appointment, or at the same appointment where possible, and given a participant information sheet and the opportunity to ask questions. Interview arrangements were made in‐person or by email or telephone.

Multidisciplinary clinician participants were key informants, identified through local clinical teams due to their ability to inform the research via professional expertise and knowledge of local HNC systems [21]. They were purposively sampled to ensure diversity of clinical roles/positions and were contacted via an email with an attached participant information sheet. Initial key informant interviews allowed for further clinical/administrative participants to be identified through a snowball strategy to obtain a breadth of perspectives. Fully informed, written consent was obtained at the time of interview for all participants.

### 2.3 Participants

There were 29 interviews undertaken in total: *n* = 17 in Glasgow (seven HNC patients, 10 professionals); and *n* = 12 in Montevideo (five HNC patients, seven professionals). Summary details of participants are shown in Tables 1 and 2. The researchers were not known to the HNC patient participants prior to the study, but were to some of the professional participants due to the highly specialized nature of the HNC teams.

**TABLE 1:** Profile of patient participants.

Location	Sex	Age (years)	Tumor site	T	N	M	Early/advanced stage TNM 8th edition
Montevideo	Male	64	Oral cavity	2	2	0	Advanced
Montevideo	Male	65	Hypopharynx	3	2	0	Advanced
Montevideo	Male	67	Oral cavity	4	0	0	Advanced
Montevideo	Female	42	Oropharynx HPV‐negative	4a	2b	0	Advanced
Montevideo	Male	69	Larynx	3	1	0	Advanced
Glasgow	Male	67	Larynx	4	0	0	Advanced
Glasgow	Female	60	Oropharynx HPV‐positive	2	2	0	Early
Glasgow	Male	61	Oropharynx HPV‐positive	3	1	0	Early
Glasgow	Male	53	Oral cavity	4	0	0	Advanced
Glasgow	Female	38	Oral cavity	3	0	0	Advanced
Glasgow	Female	61	Larynx	4	0	0	Advanced
Glasgow	Male	59	Oropharynx HPV‐positive	3	2	0	Early

**TABLE 2:** Participant role.

Participant	Montevideo, Uruguay	Glasgow, Scotland
Primary care medic	2	2
Dentist	0	2
Radiologist	0	1
Nurse specialist	0	1
Speech therapist	0	1
Surgeon	3	2
Oncologist	2	1
Head and neck service lead	1 (also oncologist)	1 (also surgeon)
HNC cancer survivor	5	7

### 2.4 Interviews

This study was undertaken over an 18‐month period from early 2022 to mid‐2023. Interviews were undertaken either face‐to‐face or online via ZOOM (Zoom Video Communications Inc. version 5.11.0) depending on participant preference and the local COVID‐19‐related mitigation measures in place at the time and given that this was deemed an appropriate software for qualitative interviews [22]. Interviews lasted on average 42 min with the longest lasting 75 min.

Interviews were undertaken by a dedicated clinical researcher in each center (G.C. and I.L.G.). They were trained in qualitative interviewing techniques by an experienced qualitative researcher (A.R.) and had experience of working with HNC patients but were not part of the centers' HNC clinical teams (G.C. is a Dental Public Health Registrar and Lecturer and I.L.G. a bilingual [Spanish and English] Oncologist who is not part of the HNC team). Interviews were undertaken at a time and location of the participants' choosing (online, a University room or local clinical setting of convenience for the participant) with support from the clinical team made available if required for HNC patient participants given the sensitive nature of the topic. Interviews were guided by topic guides that were created following a literature review and covered findings from the HEADSpAcE Benchmarking study [23].

**FIGURE 1:**
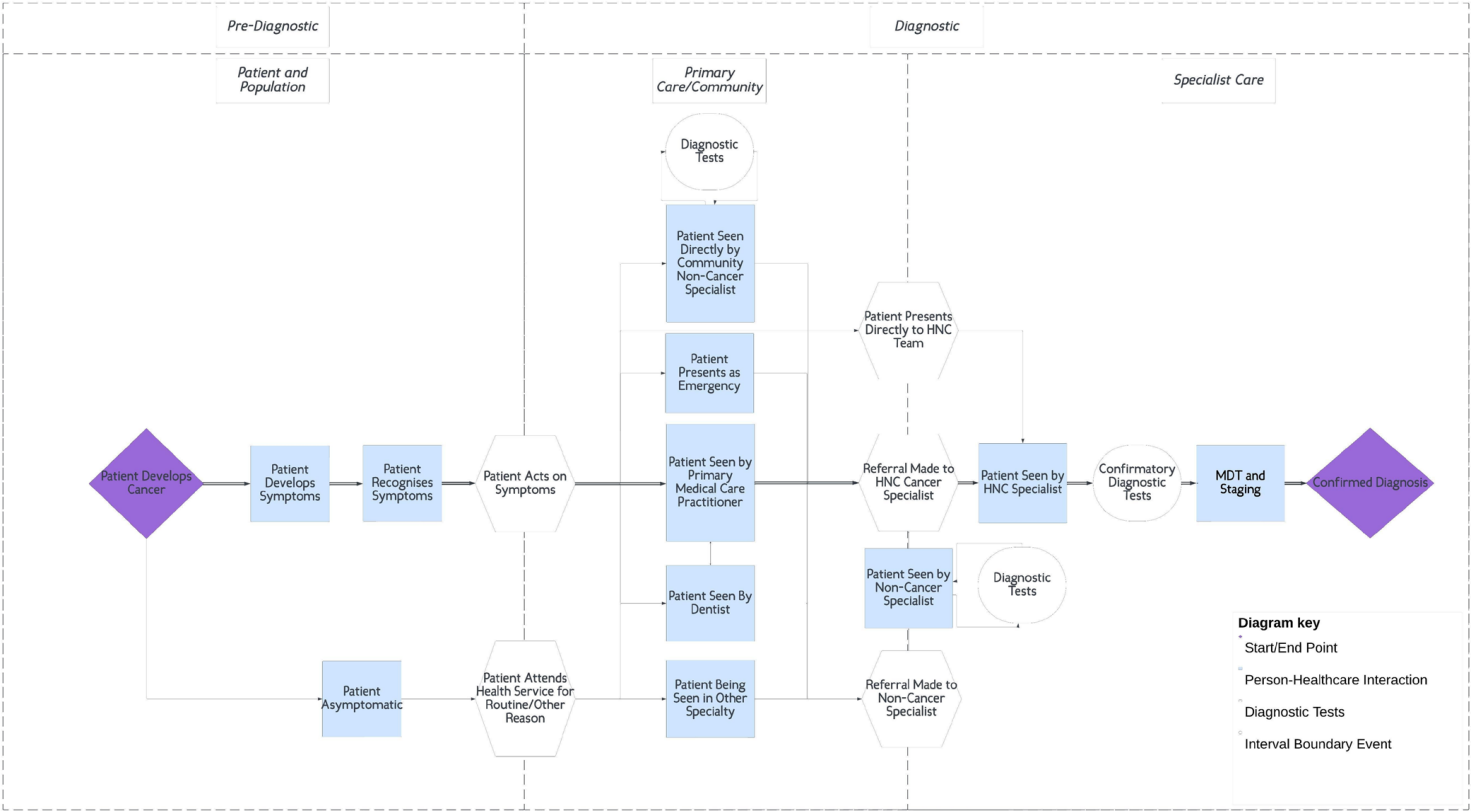
HEADSpAcE HNC diagnostic pathway. [Color figure can be viewed at wileyonlinelibrary.com]

Two templates were applied in developing interview guides and guiding interpretation: (a) the temporal frame of cancer diagnostic intervals from the Aarhus Statement on cancer diagnostic research [24] that was modified into the HEADSpAcE HNC Diagnostic Pathway in the HEADSpAcE Benchmarking study [23] (Figure 1). These intervals were the patient and population interval; the Primary and Community care Interval; and the Secondary care Interval; (b) The Systems Engineering Initiative for Patient Safety (SEIPS) model of the complex socio‐technical interactions and processes in the diagnostic journey [25]. This combination of templates allowed for probing along known diagnostic intervals and timelines and for recognized health system factors such as staff, technology, procedure, and environment.

Topic guides containing high‐level open questions with subsequent probing questions and prompts were piloted with nonparticipant clinicians in Glasgow prior to commencement of the study. Within the guides, flexibility was afforded within interviews to follow a productive line of participant response. Participants were alone apart from two whom had a partner join in the room during the interview. Single interviews were recorded for each participant except one participant who contacted the research team to clarify some of their responses and had a shorter follow‐up interview recorded (Glasgow, patient). No additional field notes were taken out with transcripts.

Each interview was recorded on an audio digital recorder or via record function on ZOOM and transcribed verbatim in the language undertaken (English in Glasgow, Spanish in Montevideo). Transcripts were checked back against original recordings to reduce transcription bias and, where necessary, were translated into English via a digital translation technology (Sonix Inc), which was then checked by the researchers (G.C., I.L.G.) for accuracy. Scripts were not returned to participants prior to analysis.

### 2.5 Analysis

Analysis was facilitated through uploading interview transcripts to Lumivero NVivo 14.0 qualitative data management software (NVivo 14 for Windows, Lumivero). Strong and meaningful concepts and subthemes that related to the aims of this study were identified through an adapted Template Analysis: a form of thematic analysis that allows interpretation of qualitative data using a priori themes (the “template” [26]) to guide interpretation while still allowing for flexibility and for subthemes and patterns to emerge.

In this study, the templates were those of the HNC diagnostic intervals and SEIPS 3.0 framework applied deductively as described above. The subtheme analysis was undertaken via a traditional inductive approach drawing from participant narratives. A sample of initial codes was developed by the primary researcher (G.C.) into provisional themes and checked independently by a senior researcher (A.R.) for consistency and quality checking. Analysis was concurrent with ongoing data collection in order to facilitate the purposive sampling of alternative perspectives and to ascertain data adequacy for the specific research aims of this study. Commensurate with the systems approach applied in this study, data collection was deemed to be sufficient upon the point of achieving data adequacy for identifying factors associated with diagnostic stage [18, 19, 27]. When no new thematic groupings were forthcoming in the interviews [28] due to the data being rich, relevant, and of sufficient quality, data adequacy was deemed to be reached for this study. Participants did not provide feedback on results before publication.

## 3 Results

Results are presented here in terms of the three main interval domains of the HNC diagnostic pathway: Patient/Population; Primary/Community Care; Specialist. There then follows brief further analysis by participant types and country/location. Finally, two linked overarching themes emerged across all intervals and participant types/locations and are detailed below: (1) the complex nature of the HNC diagnostic system; (2) the importance of communication in driving diagnostic goals within this complex landscape.

### 3.1 Interval Domains

#### 3.1.1 HNC Patient/Population Interval

The dominant themes and subthemes in this diagnostic interval pertained to patient and population HNC awareness and agency. There was consensus on the importance of improving public awareness of HNC symptoms and supporting people to navigate initial access points into the local health system when seeking to act upon symptoms:

To the patient who has a discomfort, difficulty in swallowing, consult immediately. (Montevideo, Patient)

That … that is key and that goes with early detection campaigns and going out to explain to people that if they have such and such characteristics of symptoms or signs, to consult quickly. (Montevideo, Specialist Clinician)

Many patients do not act on symptoms when they first arise and may wait for a significant time before first contacting health services. Sometimes, they are concerned the symptom might indicate cancer but can be fearful of first attendance at health services:

At the back of my mind I kept thinking, I wonder if this is cancer. I kept ignoring it. (Glasgow, Patient)

I probably knew deep down what it was, but it is quite a scary thing to face up to that. (Glasgow, Patient)

Another strong theme was the role of family and friends in prompting and facilitating these early steps in the diagnostic pathway. It was noted that friends and family can both positively and negatively impact individual decision making, with spouses/partners playing an important role in encouraging presentation to health services:

… my wife was with me and she insisted on having it done there and then and I thank her for it. (Glasgow, Patient)

It was worrisome, I consulted with my children and they decided to have me seen. (Montevideo, Patient)

This role of trust in health organizations and public bodies was an apparent issue in how people engage with health services and make decisions on accessing them:

That was subconsciously my plan, rather than going to a GP, which I don't have huge faith in. It was always my plan is to check with my colleague. (Glasgow, Patient)

I think for the better, in all aspects, improve the approach to bureaucracy… maybe today there is no doctor. (Montevideo, Patient)

One patient mentioned previous negative experience of the service as partly contributing to delay in initial access, saying

My GP [General Practitioner] … didn't give the best of treatment to my husband. (Glasgow, Patient)

There was a clear understanding, particularly, from professionals, of the role of complex social geographic and financial inequalities and how they may affect an individual's interaction with health services. This was noted as applying to HNC but also across the wider range of health issues and health services landscape. People who develop HNC disproportionately come from areas of higher socioeconomic deprivation which gives rise to complicated social and economic barriers at the community level access to health services, but also further difficulties in navigating from initial access through to specialist services:

… above all the approach to the social part of these patients, who are extremely complex and it is a deficit that we have. They are patients who come from extremely complicated backgrounds … (Montevideo, Specialist Clinician)

In terms of proposals to reduce the levels of advanced stage HNC diagnosis, there was an appreciation that public health approaches were needed for improving access to the care pathway to diagnosis. This would cover public health action to address underlying socioeconomic inequalities as well as communicating this pathway in an open way to patients.

#### 3.1.2 Primary/Community Care Interval

Knowing which health service to attend in the first instance for symptoms of potential HNC was an issue, highlighting overlapping roles of dentists and general medical practitioners in this role in the system:

… patients are maybe phoning their doctor for things to do with their mouth but there's been things to do with like throat or maybe like neck and things and they're told to come to their dentist. (Glasgow, Primary Care Clinician)

Dentists do not refer anywhere … they say ‘I have this patient, I am going to refer him to you’ and then we (general medical practitioner) are the ones who make the referral. (Montevideo, Primary Care Clinician)

Once individuals have made the decision to contact and engage with health services there were several important themes. There was a clear expression that HNC is a poorly recognized and understood disease at the primary care level, particularly, in primary medical care, as the symptoms are often potentially attributed to other diseases that are less serious and more routinely seen. There were many strong expressions of reported misdiagnosis, including several repeat prescriptions for antibiotics when patients attended primary care:

From the moment they consult with their first symptom, go through a series of general practitioners who give them different treatments, antibiotics, painkillers. (Montevideo, Specialist Clinician)

I finished the course of antibiotics and then went back again and then got another set of antibiotics. (Glasgow, Patient)

Not having standardized equipment, such as scopes to visualize the oropharynx, was said to be a barrier in examining in primary care, which points to a desire for an enhanced diagnostic role in primary/community care:

Yes, there are things … Not all places have the equipment, such as endoscopes, to make a diagnosis. (Montevideo, Specialist Clinician)

There is a reported need for training and education for primary care practitioners on HNC and how it presents, symptoms and signs, and on the criteria for urgent referral for suspected cancer. It was suggested the training should be delivered by the specialists from HNC treatment centers, which would also improve primary care and specialist engagement:

Obviously there is always room for improvement, and I think it would be another point that would be good to think of improvements in terms of training for everyone and on a more frequent basis. (Montevideo, Specialist Clinician)

I think that there is a lot of potential I would imagine in increased awareness among GPs. (Glasgow, Specialist Clinician)

There were also reflections on options for screening of HNC targeted to those highest risk (those with traditional smoking/alcohol risk factors and or from particular areas/backgrounds) although there was acknowledgement that there was a need to build the evidence base for a formal screening program:

There is no way to see that without a clinic exam. So that would be the part of screening that you would need if you were doing it clinically … there are blood tests and things that are currently under research … I think it would have to be extremely targeted. (Glasgow, Specialist Clinician)

I have been taking part in some sort of screening events over the years where most of the stuff you see is benign, so I'm not quite sure how effective it is. (Glasgow, Specialist Clinician)

Finally, the issue of honest, frank communication was noted. Patients and professionals felt that the risk of developing HNC was not explicitly discussed during healthcare appointments prior to diagnosis and there were potential missed opportunities for interventional discussions/referrals to services that may support modifying certain lifestyle behaviors such as smoking and/or alcohol consumption, relating to the theme of trust presented earlier:

Maybe a bit more forthrightness with each other. Sometimes we do things, and we don't want to hurt people's feelings …(Glasgow, Specialist Clinician)

… if the dentist would be more forthcoming with a wee bit of information about what could potentially happen. (Glasgow, Patient)

Communication to clinical teams in primary and secondary care was also proposed as beneficial as was developing data monitoring capability and harnessing existing data:

We have improved a lot and we have a very good cancer registry because in order to make health policy on cancer you have to have and know what the incidence and mortality in this case of localization is. (Montevideo, Specialist Clinician)

#### 3.1.3 Specialist Services Interval

The main themes expressed related to the specialist services interval were related to referral processes/systems. There was an expressed need for formalized referral systems that were open to wider health teams, not only primary care medical practitioners:

for people presenting with concerning signs to community pharmacies … make sure that they have an established pathway to report their concerns. (Glasgow, Primary Care Clinician)

In addition, there were calls for clearer, more accessible referral guidance to enable the referral pathway to be better utilized. There were frustrations at the heavy volumes of bureaucracy which impact efficiency and the referral processes particularly when it comes to the ability to share clear information, such as having to include information that is already available through medical records and not being able to easily upload photographs to referrals. Clarity of information was seen as key for reducing any potential delays in referral processing:

They have to be quite clear and concise of all the symptoms. (Glasgow, Specialist Clinician)

The benefits of referral triaging and time monitoring for ensuring that appropriate patients are seen as quickly as possible were also expressed:

I believe that in this sense, some kind of articulating personnel between both levels would be very useful who could filter, to some extent triage, the patients who have some element of organicity and who require a quicker consultation. (Montevideo, Specialist Clinician)

These referral aspects were part of a wider disconnect between primary care‐and specialist services where the health system for the pathway to HNC diagnosis is seen as challenging for primary care professionals as specialist services are the sole gatekeepers for cancer diagnosis and there is no shared ownership over the diagnostic pathways:

I believe that there is a devaluation of the first level of care physicians, there is a devaluation of family and community medicine … (Montevideo, Primary Care Clinician)

Potentially … unintentionally and subconsciously having a change or shift in referrers' behaviours so that gatekeeping is essentially being moved into secondary care where they have, I am pretty sure, unintentionally taken on that role of gatekeeping. (Glasgow, Primary Care Clinician)

It was also suggested that patients could have their journeys to diagnosis better supported and facilitated to counteract some of those earlier expressed complexities. This could for example be done through the role of a patient coordinator/facilitator for those being referred for suspected HNC:

There has to be a nurse in charge of coordinating everything for the patient or someone who coordinates everything. (Montevideo, Specialist Clinician)

… a patient navigator essentially to try and make sure that people are accepting the earlier scans and everything that they can. (Glasgow, Primary Care Clinician)

Similarly to primary care, the availability of resources for undertaking and reporting specialist diagnostic investigations was considered essential in facilitating early diagnosis. There was also the suggestion of increasing access to histopathology and radiology services directly from primary care/community care:

I think if you had your radiology and scanners and all these types of things not necessarily in GP practices, but in community‐based healthcare facilities, you might get somewhere. (Glasgow, Primary Care Clinician)

The importance of having immediate investigations without delays following presentation to specialist services was emphasized; with several diagnostic improvements to the process for requesting and automating some aspects of this process rather than relying on human prompts:

… it would be lovely if there was one unified request, that I could move that patient along the system … I should just be able to press a button and move that sequentially along the system. (Glasgow, Specialist Clinician)

More broadly, there were reported challenges in terms of demand on the overburdened health systems and availability of HNC specialists. On occasion this had impacted where specialist clinicians would bypass existing systems to get things done:

Capacity is the main barrier, yes, without a doubt yes. (Glasgow, Specialist Clinician)

We try to expedite … I give them the phone number so that they can go to the consultation or consult me. (Montevideo, Specialist Clinician)

There was also the expressed wish for utilizing a wider workforce skill mix and the diagnostic care pathway so that specialists/senior staff are not overly burdened with tasks that could be readily undertaken by other specialists:

The specialist does not fulfil the function of a specialist only … have to do research, to do epidemiology or to do social work. (Montevideo, Specialist Clinician)

Regarding HNC service structure and design, there were conflicting suggestions as to the utility of centralisation, with specialists considering a centralized service as the gold standard as it meant better access to full multidisciplinary team, yet challenging in terms of physical access to the service and in relation to geographic inequalities:

I think it's, I guess, quite a good thing to have it all centralised because then if it's centralised everyone knows everyone. (Glasgow, Specialist Clinician)

… we also have to take into account the distances in which each of the people live and where they must be attended. (Montevideo, Primary Care Clinician)

There were additionally conflicting opinions on the role of the multidisciplinary team whereby it may improve treatment delivery but may not have any positive impact on the stage of diagnosis:

…as a specialist looking after a patient you probably feel less able to make your own decisions and get the ball rolling …. (Glasgow, Specialist Clinician)

in terms of then getting through the pathway, I do think there are ways that we can probably make that quicker … the meeting is once a week and often we find that nothing gets done between the meetings. (Glasgow, Specialist Clinician)

Finally, the role of technology and diagnosis on improving communication in the HNC diagnostic pathway was highlighted, including the potential of artificial intelligence aiding diagnostic reporting.

if we could have more technology it would make it much easier for, for those coming up the learning ladder to see better and learn better. (Montevideo, Specialist Clinician)

There was some caution from a patient perspective given in relation to some technological approaches such as difficulties with remote online consultations and how they could contribute toward misdiagnosis:

I mean how can you make a diagnosis over a telephone call? (Glasgow, Patient)

### 3.2 Overarching Themes Across the Diagnostic Pathway

#### 3.2.1 Communication

Across all aspects of the diagnostic pathway (interval domains) the main overarching theme was that of communication and information transfer issues and barriers both between health service and patients and also between components of the health system/services. Achieving accurate and timely diagnostic outcomes through this dynamic work and community system substantially relies upon communication, which must be optimized through formal stakeholder links and through nurturing interpersonal relationships with trusted partners:

I believe that the links with second level specialists [and primary care teams] need to be improved. That would go a long way toward shortening times and improving patient care. (Montevideo, Primary Care Clinician)

Well, communication, I think, is fundamental in these patients and it is also fundamental to personalize it. (Montevideo, Specialist Clinician)

Many of the communication barriers described related to the role of processes and technologies including difficulties accessing and sharing relevant information and this issue was, particularly, relevant to the referral processes from primary care into specialist services:

I think pathways between us can be much, much better. Quite often professionals will just hang up the phone and can't wait for ten minutes for somebody to answer the phone. Quite right, I wouldn't do either. (Glasgow, Specialist Clinician)

It seems to me this whole referral system is set up to suit secondary care and certainly not to suit primary care and not to suit the patient. The patient journey is non‐existent with their referral mechanisms. (Glasgow, Primary Care Clinician)

Additionally, due to the complex and untransparent nature of the diagnostic pathway, there were several perceived issues with communicating with HNC patients on how best to navigate that pathway with an associated need to do more in public health terms to communicate risk with HNC patients who have traditional behavioral and socioeconomic risk factors for developing HNC. This of course illustrates another complexity of the system in that the outcomes emerge from interactions with these community factors.

#### 3.2.2 The Complex Head and Neck Diagnostic System

The application of the SEIPS systems model illustrates the complex nature of the diagnostic landscape that patients and staff have to negotiate and navigate, with these thematic results naturally sitting within the SEIPS 3.0 domains (Figure S1). The work system can be conceptualized as a collection of elements: persons with roles and responsibilities (e.g., patients, oncologist, PCPs), performing tasks (e.g., ordering a test), while using various tools/technologies (e.g., electronic health records, human resource, decision support), working in an environment (e.g., office, clinician workroom, home), and sitting in an organizational context (e.g., practice, guidance, staffing). There are professional aspects (e.g., primary care using guidance to inform clinical decision making, communication between clinicians), patient aspects (e.g., management of activity and diet as a contribution of health maintenance, attending services, undergoing recommended opportunistic cancer screening) and shared work processes (e.g., shared decision making between clinician and patient). Often, this system requires tasks that may not be entirely delineated and may be shared and equally actionable between multiple individuals.

It can be observed from patient and staff interviews that HNC patients interact with multiple health system elements and external environments in their nonlinear pathways to diagnosis.

To me, the system of having this to do that, to do that, to do that, is putting too many obstacles in the way of seeing someone who knows what they're talking about from day one, right at the beginning. (Glasgow, Patient)

Family, community and work influences, including geographical and financial inequalities, set norms and form dynamic initial conditions through which health issues and symptoms are experienced and understood, and engagement with the health system is initiated (or otherwise).

Pains appear, symptoms appear and suddenly you assimilate them because of the experience you have from other people or from relatives or acquaintances. (Montevideo, Patient)

Service access and engagement is complex due to a number of interactions including: knowledge of appropriate services; unclear responsibilities of medical and dental professionals in primary care; emergent risk from a lack of professional case‐based experience and/or training; and difficult screening decisions including efficiency‐thoroughness trade‐offs.

I see probably about one a month where they tell me that they saw their GP with throat pain and it was dismissed. Or it was reassured that the pain didn't get better and then they went back and they got some antibiotics and maybe another course of antibiotics. Then when you see them there is nothing you can do there. (Glasgow, Specialist Clinician)

There is further complexity in referral and investigative processes which bring in some further context in tools and technologies, tasks and the organization of work, for example: staffing pressures which might indicate benefit in the use of the wider skills of the team; lack of clear guidance for efficient referral is a known issue; the need for data and optimal governance, monitoring and oversight; specialist investigations are resource‐pressured and could benefit from widening staff access and incorporating better feedback and feedforward loops.

I think that the bureaucracy, the bureaucracy of public health and of each institution does not work in a coordinated way, there is no smooth coordination. (Montevideo, Specialist Clinician)

### 3.3 Interpretation by Participant Type and Location

#### 3.3.1 Differences Between Glasgow and Montevideo

A key difference between sites was the overall sense of the main contributors toward the high levels of advanced stage diagnosis. In Glasgow, the general feeling was that the major issues were in initial access to primary care, accurate initial detection, and in the process of referral to specialist services. However, in Montevideo it was felt that there were significant issues in the specialist services themselves, including in the organization and structure of specialist clinics (and availability of specialist services more generally).

Another key difference was the availability of and access to diagnostic investigations. In Glasgow, it was noted that these happened rapidly once an HNC patient was referred. However, in Montevideo there were significant bureaucratic and human resource limitations (resulting in long waits for pathology, in particular).

With regards to referral systems, in Montevideo there was an expressed need for referral guidance and clear protocols whereas these existed in Glasgow at the time of the study. The feeling and experiences of those in Glasgow were that the guidance was useful but not well utilized and that there was still a need for training, particularly, of primary care clinicians. Glasgow already has in place a bespoke electronic referral system for suspected patients with HNC; however, there were significant frustrations expressed particularly by primary care clinicians of both the bureaucracy and operational effectiveness of the system. In Montevideo, clinicians expressed a strong need for an electronic referral process system for patients with suspected HNC.

Other themes expressed solely in Montevideo were the significant travel distances experienced by patients in accessing specialist care which did not exist in Glasgow to the same extent. In Glasgow, but not in Montevideo, there was explicit discussion given to the important role of dentists and how they integrate into health systems. Additionally in Glasgow there was attention given to the increasing incidence of HNC and to the role of HPV and changing the profile of HNC (i.e., more oropharyngeal cancer cases), which would impact on both specialist care services and potentially on referral pathways.

#### 3.3.2 Differences Between Participant Types

More differences were observed between patients and clinicians, as might be expected given the experience—those going through the diagnostic pathway compared to those delivering it—and knowledge—expertise in different areas of the intervals and the HNC systems.

Patient participants were forthright in their views over the lack of explicit cancer discussions and risk factors in their early interactions with health services prior to their diagnosis. Patient participants generally feel the diagnostic pathway is longer and slower from start to finish than professionals do, and stress the difficulties of multiple healthcare visits and navigating challenging barriers to access the right services. Patients generally stressed the importance of the role of family and friends in prompting initial attendance to health services and supporting them through diagnosis. Patients also shared that their perception of the severity and importance of symptoms played into how they assessed when they should contact health services. Almost unanimously, patients did not immediately act on the initial health concern because they didn't think it was significant.

A key theme to come from the patient participants that was not fully appreciated by the clinician participants was that of trust and attitude toward health organizations and authority more generally. This was evident across patient interviews where an existing distrust of our previous negative experience was a barrier to them engaging with health services promptly.

For professional participants the key issues were much more in the processes of the health system pathways to diagnosis, particularly, with regards to how patients go through the health system from primary care to specialist services and are provided with specialist diagnostic investigations. Much comment was made on the organization of clinics and having all equipment and staffing resource available. Clinicians expressed the need for a national strategy including clear protocols and harnessing data to improve services, in addition to improving HNC population knowledge through improved communication strategies targeted to both the general population and primary care clinicians.

## 4 Discussion

To our knowledge, this is the first study to investigate barriers and delays in the diagnostic pathway for HNC through a health systems approach using qualitative methods with patients and staff. We aimed to understand health system factors associated with advanced stage HNC diagnosis in two centers embedded in our international HNC consortium. The reason for selecting two high income countries was to limit the potential impact of the wider socioeconomic context, to some degree, and to enable the focus on local/center level health system factors. We interviewed both patients and multidisciplinary clinicians to ascertain their perspectives on HNC diagnostic pathways. We identified the key themes of population knowledge of HNC and agency in navigating health systems, the role of family members, socioeconomic, and geographic inequalities, the disconnect between primary and secondary care, and professional perspectives on complex interacting working elements.

There were a number of strengths to this study, including obtaining a whole‐systems view to the HNC diagnostic pathway through including patients and a range of clinicians as participants. Additionally, undertaking this study in multiple centers gives a unique insight into some of the findings that would not be as key if just one center was included. Applying the findings to the framework of SEIPS 3.0 helps form a system level interpretation of results to facilitate potential intervention to optimize this complex and important health pathway.

This study adds to the existing literature on factors associated with stage of diagnosis and HNC, highlighting for the first time the role that health systems play in addition to patient related factors. Previously the emphasis has been placed on patients in these time intervals to diagnosis, defined pejoratively as patient delays [29]. However, when considered as part of the health system, there is responsibility for organizations to consider patient agency and ease of access to services, as well as how smoothly these systems can be navigated through until the point of diagnosis of HNC. Our findings suggest that the issues in the patient interval are more socio‐organizational rather than individual (Figure S1). This study is innovative in combining the structural model of the diagnostic pathway with the systems lens of SEIPS which is a validated theory based tool embedded within healthcare systems, including the NHS in the UK [30].

Limitations to this study include that only patients with HNCs who had survived treatment were able to be interviewed given the timings of receiving a diagnosis, starting treatment and being fit enough to participate in an interview. This means that HNC diagnostic pathways for patients who did not survive or were on a palliative care pathway were not included; however, it is likely that many of the findings would apply. Another limitation is that interviews were carried out in the common language of the center, Spanish in Montevideo and English in Glasgow. This means that potentially important factor in HNC patient ability to navigate health systems, the role of language barriers, which can be an access issue in accessing healthcare and a factor in poor health outcomes [31], was not covered in additional depth. Interventions to mitigate language barriers in research have been well evidenced in existing literature [32], which could be applied to HNC. Access to potential participants was made more challenging due to the COVID‐19 pandemic and associated mitigations that came just as this study was being conducted in the early stages, resulting in challenges in study approvals and recruitment of participants.

This study has obtained a systems‐based, holistic view of the HNC diagnostic pathway; further research focussing specifically on the possible variation in experiences of patients with early and advanced disease would be beneficial. By having two centers that are publicly funded in a similar fashion, we enabled comparisons to be made; however, a further limitation is that we were unable to include participants from health systems in low‐middle income countries or those that are funded through different models, meaning that the factors associated with advanced‐stage diagnosis may differ across different types of health system.

This study correlates with findings from the HEADSpAcE Benchmarking study [23] in several important ways. Firstly, the expression in this study that socioeconomic factors play a key role in stage of HNC diagnosis is linked to the role of having a publicly‐funded health system which aims to mitigate the role of financial costs in navigating the pathway to HNC diagnosis. Additionally, having a formalized referral triaging system and routinely monitoring referral waiting times relates to the themes here of utilizing data and formalizing referral pathways.

Improving population knowledge and awareness of HNC has been described as a key factor in reducing advanced stage diagnosis [33, 34], although the literature is conflicted as previous evidence shows there is no impact on stage [35]. Our findings suggest that knowledge and awareness of HNC alone are perhaps insufficient and we were able to be more specific here in that there is a need for health literacy and agency in accessing and navigating health services through the diagnostic pathway. More than knowledge and awareness, there were proposals for facilitating/supporting patients through the diagnostic care pathway with referral coordinator/support worker to help navigate the seemingly complex system.

Additionally, the utility of referral guidance/protocols is also an interesting finding. Even in Glasgow, where such guidance exists in the form of the Scottish Cancer Referral Guidance that provides primary care clinicians with clear thresholds that would indicate the need for a referral for an urgent suspicion of cancer [36], some clinician participants were not aware of them. Studies have shown that although guidance has an important role to play, it must be implemented/utilized and fully communicated in order to have the desired aim of improving the referral/diagnostic pathway [37]. The other difference noted between sites, the role of dentists in this pathway, is likely a reflection of the difference in how dentistry is incorporated into wider health systems. In the United Kingdom, dentistry is a core part of primary care in the NHS with services designed to be available to all, whereas in Uruguay, the publicly available element of dental services is more limited.

Despite some participants calling for HNC screening programs, there is insufficient evidence to support formal screening programs for HNC's. There is some evidence recognizing the role of opportunistic screening by health professionals, particularly dentists, for detecting oral potentially malignant disorders or early stage oral cavity cancer via routine clinical oral examinations [38]. As there are wide socioeconomic inequalities in the burden and outcomes of HNC [39, 40, 41], “upstream” policy‐level changes will be essential to address these inequalities and reduce the population disease burden.

Our findings of a disconnect between primary care and specialist care, trust in clinicians, and limited knowledge/lack of guidelines correlates with that of a large systematic review from 2016 across the cancer spectrum [42]. The finding here of the blurring of the role of dentists and medics in primary care also correlates with the results of a 2019 systematic review which found variance between both sets of professionals with regard to HNC referral [37].

The role of modern technologies and processes involved in the diagnostic HNC patient journey may also play a key role in prompt diagnosis and early detection of HNCs. The emergence of telemedicine has been proposed as a possible route to improve early diagnosis rates and the use of modern electronic HNC patient records can improve inter‐professional workflow, reducing friction and delay in the HNC patient journey, and even predict future diagnosis by identifying changes in healthcare use [43, 44].

## 5 Conclusions

This study offers, from both patient and clinician perspectives, a deep insight into health system factors associated with the HNC diagnostic pathways leading to advanced stage disease in two centers that are part of the HEADSpAcE Consortium, Glasgow, Scotland, and Montevideo, Uruguay. There are key themes pertaining to all areas of the referral to diagnosis pathway, including the ability of individuals to access and navigate health services, the structure and organization of health services, and communication between key people in the diagnostic journey to HNC diagnosis that could prove beneficial for future research areas and developing interventions to reduce the burden of advanced stage HNC.

### 5.1 Reflexivity

The primary researcher in this study, GC, is a dentist and registrar in dental public health with experience in the direct care of patients with HNC from referral through to patient care in primary care and specialist services in the United Kingdom and New Zealand. They have personal experience of a close relation who was diagnosed with cancer (non‐HNC) at Stage IV as a young adult. Patient participants were not explicitly made aware of this latter fact unless it came up in discussion at interview time. I.L.G. is an Oncologist, but is not part of an HNC team. Potential impacts of the professional roles in interview consistency were mitigated through the use of standardized topic guides and regular discussions following interviews on findings to help achieve data adequacy.

## Author Contributions

Study conceptualization was performed by G.C., A.R., D.I.C., S.V., and P.B. Clinical guidance was provided by C.P., M.C., and J.M. Recruitment was carried out by G.C. and I.L.G. Data quality checking was conducted by G.C. and I.L.G. Analysis was performed by G.C., I.L.G., and A.R. Manuscript drafting was completed by G.C. All authors contributed to the manuscript and participated in its revision.

## Funding

This study was funded by EU Horizon 2020 Framework Programme Grant Agreement No 825771.

## Disclosure

Where authors are identified as personnel of the International Agency for Research on Cancer/World Health Organization, the authors alone are responsible for the views expressed in this article and they do not necessarily represent the decisions, policy or views of the International Agency for Research on Cancer/World Health Organization.

## Ethics Statement

This study was approved by local ethical approval including from the NHS WOSREC 3 21/WS/0038.

## Consent

All participants provided informed consent at the time of interview.

## Conflicts of Interest

The authors declare no conflicts of interest.

## Supporting information


**Figure S1:** Results of thematic analysis presented by diagnostic interval and SEIPS 3.0 domain.

## Data Availability

The data that support the findings of this study may be available on request from the corresponding author. The data are not publicly available due to privacy or ethical restrictions.
