# Supplementary material for: Health System Factors in Head and Neck Cancer Diagnosis: A HEADSpAcE Consortium Qualitative Study in Glasgow and Montevideo
Source: Head Neck. 2026 May 10;48(10):2849–62. doi: 10.1002/hed.70262 (PMC13595473; doi:10.1002/hed.70262)
Supplement: Supplementary file 1 [file HED-48-2849-s001.pdf]

System Factors in Advanced Stage Diagnosis of Head and Neck Cancer: A HEADSpAcE Qualitative Study Across Glasgow, UK, and Montevideo, Uruguay.

HEADSpAcE

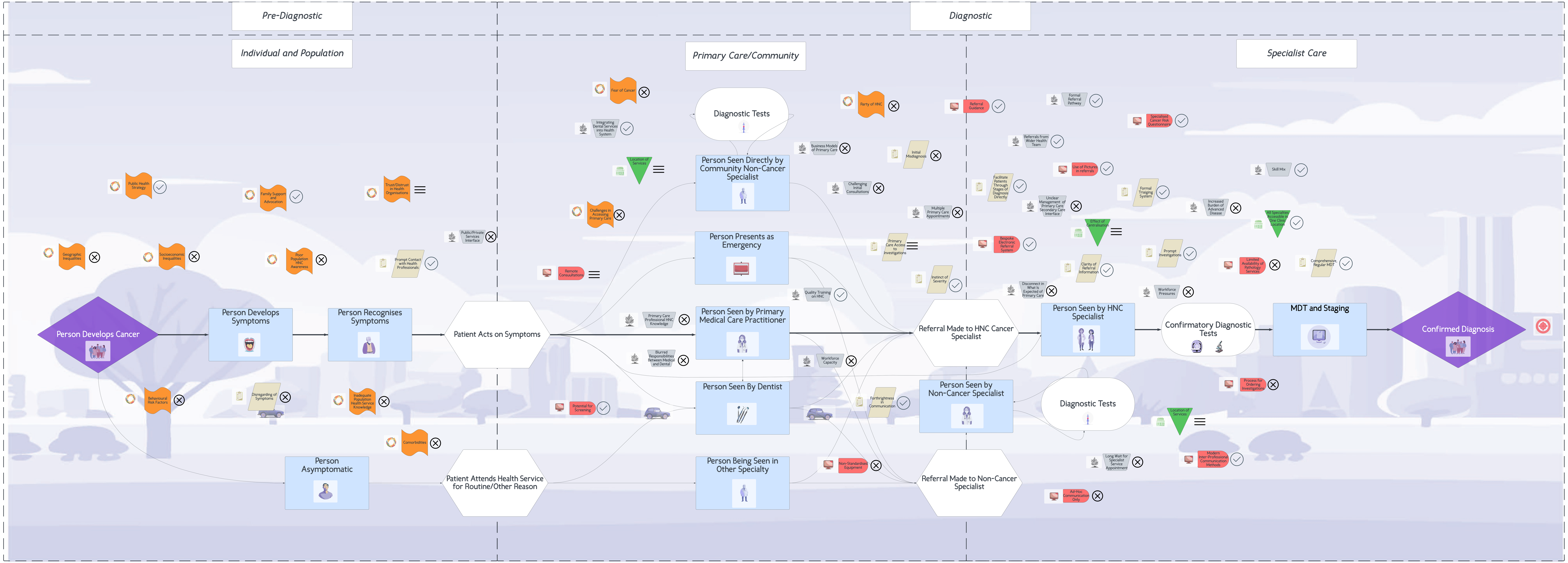

This diagram is a product of the Qualitative Study involving Semi-Structured Interviews with key stakeholders, incuding patient survivors, acrsoss two Head and Neck Cancer (HNC) centres in the HEADSpAcE Consortium: Glasgow, UK and Montevideo, Glasgow. The results were thematically analysed following a template analysis using a modified version of the Aarhus cancer statement (created following the HEADSpAcE Benchmarking Study which ran in parallel with this study) to define the chronological steps involved in diagnosing HNC. Subsequently, the themes were then applied to the domains of the SEIPS 3.0 model of systems enginering in health services pertaining to patient diagnostic journeys to reflect what the potential barriers to and facilitators of early HNC diagnosis are and where they lie in the context of the HNC diagnostic athway. This helps visualise the potential issues existing and helps focus further research and intervention ideas.

Pascale Carayon, Abigail Wooldridge, Peter Hoonakker, Ann Schoofs Hundt, Michelle M. Kelly, SEIPS 3.0: Human-centered design of the patient journey for patient safety, Applied Ergonomics, Volume 84,2020, 103033, ISSN 0003-6870, doi.org/10.1016/j.apergo.2019.103033.
Weller, D., Vedsted, P., Rubin, G. *et al*. The Aarhus statement: improving design and reporting of studies on early cancer diagnosis. *Br J Cancer* 106, 1262–1267 (2012). https://doi.org/10.1038/bjc.2012.68

Diagram Key for Patient Journey and Interactions with Staff

- Person-Healthcare Interactions
- Diagnostic Tests
- Interval Boundary Event
- Start/End Point

Diagram Key for System Factor Classification

- Tools and Technology
- Tasks
- Socio-Organisational Context
- Organisational Conditions
- Physical Environment
- Facilitator to Early Diagnosis
- Mixed Barrier-Facilitator
- Barrier to Early Diagnosis
